# Transcriptomic and machine learning analyses identify hub genes of metabolism and host immune response that are associated with the progression of breast capsular contracture

**DOI:** 10.1016/j.gendis.2023.101087

**Published:** 2023-09-09

**Authors:** Yukun Mao, Xueying Hou, Su Fu, Jie Luan

**Affiliations:** Breast Plastic and Reconstructive Surgery Center, Plastic Surgery Hospital, Chinese Academy of Medical Sciences, Peking Union Medical College, Beijing 100144, China

**Keywords:** Biomarker, Breast capsular contracture, Immune infiltration, Machine learning algorithms, PRKAR2B

## Abstract

Capsular contracture is a prevalent and severe complication that affects the postoperative outcomes of patients who receive silicone breast implants. At present, prosthesis replacement is the major treatment for capsular contracture after both breast augmentation procedures and breast reconstruction following breast cancer surgery. However, the mechanism(s) underlying breast capsular contracture remains unclear. This study aimed to identify the biological features of breast capsular contracture and reveal the potential underlying mechanism using RNA sequencing. Sample tissues from 12 female patients (15 breast capsules) were divided into low capsular contracture (LCC) and high capsular contracture (HCC) groups based on the Baker grades. Subsequently, 41 lipid metabolism-related genes were identified through enrichment analysis, and three of these genes were identified as candidate genes by SVM-RFE and LASSO algorithms. We then compared the proportions of the 22 types of immune cells between the LCC and HCC groups using a CIBERSORT analysis and explored the correlation between the candidate hub features and immune cells. Notably, PRKAR2B was positively correlated with the differentially clustered immune cells, which were M1 macrophages and follicular helper T cells (area under the ROC = 0.786). In addition, the expression of PRKAR2B at the mRNA or protein level was lower in the HCC group than in the LCC group. Potential molecular mechanisms were identified based on the expression levels in the high and low PRKAR2B groups. Our findings indicate that PRKAR2B is a novel diagnostic biomarker for breast capsular contracture and might also influence the grade and progression of capsular contracture.

## Introduction

Silicone breast implant surgery is the prevailing method of breast reconstruction and repair performed after the surgical treatment of breast cancer.^1^^,^^2^ Owing to their realistic feel, cosmetic advantages, and the safety of the surgery, silicone breast implants have been popular since their introduction. However, postoperative complications are not uncommon, and one of the most common problems after breast augmentation surgery/breast reconstruction post breast cancer surgery treatment is capsular contracture. The incidence rate of capsular contracture ranges from 2.8% to 20.4%.^3^^,^^4^ After the silicone is implanted, a layer of capsule is usually formed on the surface. This normal fibrotic foreign body reaction typically resolves on its own, as it is a self-limiting process. However, when the capsule is too thick or the contractive force is too strong, breast pain and even deformity can occur.^5^^,^^6^

In clinical practice, the extent of capsular contracture is commonly categorized into four grades based on Baker's grading system.^6^ Grade I breasts are soft without abnormalities; grade II breasts are slightly firm and the implants are palpable but invisible; grade III breasts are moderately firm and the implants are palpable and visible; and grade IV breasts are extremely firm with implant distortion and severe pain.^6^ At present, the Baker grading involves the grading of clinical symptoms, which is a qualitative evaluation that is affected by the judgment of the plastic surgeon.

In recent years, research has focused on identifying indicators for breast capsular contracture to more accurately diagnose or grade the condition. Some studies suggested that capsular contracture is related to biofilm formation and can be diagnosed by analyzing the bacterial microbiome.^7^^,^^8^ However, María Guembe's team^9^ demonstrated that capsular contracture was associated with neither high biofilm production nor breast implant colonization. According to Zambrano et al,^10^ ultrasound is a valuable technique that can be used to assess various aspects of the capsular contracture. Lee's team^11^ reported that the severity of breast capsular contracture might be assessed simply by evaluating the thickness of the capsule. Capsular contracture has also been observed by histological analysis, and the alignment of collagen fibers was found to vary between non-contracted and contracted capsules. Additionally, the capsules from patients with contracture categorized as higher Baker grades were thicker and exhibited greater levels of tissue inflammation.^12^ Microstructural examinations revealed that silicone capsules had a multi-layered structure, with an inner layer characterized by synovial-like metaplasia. This inner layer was more prevalent in healthy uncontracted capsules.^13^^,^^14^

Based on the aforementioned findings, it is evident that there is a lack of consensus regarding the diagnostic and grading criteria that should be used to assess breast capsular contracture. Consequently, there is a need to identify quantitative indicators and potential underlying mechanism(s) to permit a more accurate diagnosis and treatment of breast capsular contracture. In the present study, we aimed to identify hub features for breast capsular contracture by using RNA sequencing technology^15^ combined with machine learning algorithms.^16^^,^^17^ We also predicted the potential mechanisms underlying breast capsular contracture. Furthermore, as capsular contracture was recently reported to be related to a macrophage-mediated chronic inflammatory response,^18^ we used the CIBERSORT database to assess the potential relationship between infiltrating immune cells and the diagnostic features of the condition.

## Materials and methods

### Collection of samples and patient information

We obtained breast implant capsule samples from a total of 12 female patients (15 breast capsules) who underwent breast implant removal/exchange surgery at the Breast Plastic and Reconstructive Surgery Center, affiliated with the Plastic Surgery Hospital, Chinese Academy of Medical Sciences, during the period between January 2022 and January 2023. Ethical approval for this study was obtained from the Ethics Committee of the Plastic Surgery Hospital. The severity of capsular contracture was assessed using the Baker grading system. The clinical characteristics of the patients are summarized in Table S1. Breast implant capsule tissues from patients classified as Baker grade I or II were categorized as the low capsular contracture (LCC) group, representing relatively normal capsules. On the other hand, breast implant capsule tissues from patients with Baker grade III or IV were assigned to the high capsular contracture (HCC) group, representing more severe capsular contracture. All breast implant capsule samples were stored at −80 °C until they were analyzed.

### RNA extraction

The SMART-Seq® HT Kit was utilized to extract total RNA from the breast implant capsule samples, following the instructions provided by the manufacturer. The quality and integrity of the extracted RNA were assessed using a bioanalyzer from Agilent Technologies (US). The extracted RNA was subsequently utilized for further studies.

### Microarray assays

To construct RNA libraries, the VAHTS Ribo off rRNA depletion kit (Vazyme, N406-02) was used to deplete rRNA, and then the rRNA-depleted RNA was fragmented and transformed into double-stranded complementary DNA (cDNA). A cDNA library was created using the VATHS University V6 RNA seq library preparation kit (Vazyme, NR604-02) for Illumina. The library was checked using a Qubit fluorometer and an Agilent 4200, followed by sequencing on the Illumina NovaSeq 6000 sequence platform (selecting the PE150 mode). The differential expression analysis was conducted using the “limma” package, considering genes with a | log_2_(fold change) | > 1 and *P*-value <0.05 to be significant.

### qRT-PCR

The concentration of RNA was measured using a NanoDrop 2000C ultradifferential spectrophotometer. Subsequently, RNA was reverse-transcribed into cDNA using the HiFiScript cDNA synthesis kit according to the manufacturer's instructions (CoWin Biosciences, Beijing, China). The mRNA expression levels were analyzed using the SYBR FAST qPCR kit Master Mix (KAPA Biosystems, Wilmington, USA). The primers for qPCR were designed using Primer3 Plus (Table S2). GAPDH was utilized as a reference gene. The expression of PRKAR2B in all capsular contracture samples was determined by the 2^−ΔΔCT^ method.

### Western blot analysis

Capsule tissues were extracted using pre-cooled RIPA buffer supplemented with a cocktail of protease inhibitors from Roche to extract the total protein. Following 30-min incubation on ice, the protein lysates were centrifuged and the protein concentration was determined using the BCA Protein Assay Kit from cwbiotech. Prepared proteins were loaded onto a polyacrylamide gel for electrophoresis to separate the proteins. Then, proteins were transferred to polyvinylidene fluoride (PVDF) membranes, which were blocked with a BSA solution. Membranes were then washed and incubated with primary antibodies against PRKAR2B or β-actin (both from Abcam; both 1:1000). Following three washes, the membranes were then incubated with anti-mouse or anti-rabbit secondary antibodies, and the band intensity on the membranes was quantified using the ImageJ software. The protein levels of PRKAR2B were normalized to those of the housekeeping protein, β-actin, which served as a control.

### Identification of hub genes for capsular contracture

To investigate hub genes, the least absolute shrinkage and selection operator (LASSO) and Support Vector Machine (SVM) machine learning algorithms were utilized. These algorithms were used to analyze the data and identify potential genes with prognostic significance. The SVM model was established using the “e1071” software package. To reduce dimensionality, the glmnet package was used to implement the LASSO method. This approach helped us select the most relevant features for our analysis. The overlapping genes were obtained from learning algorithms and were considered hub features. The diagnostic relevance of hub features was assessed using the “pROC” package, which enabled the construction of receiver operating characteristic (ROC) curves. The diagnostic efficacy of prognostic genes in the context of capsular contracture was assessed based on the area under the curve (AUC), where a larger AUC value indicates a higher diagnostic value.

### Functional enrichment analysis

The Gene Ontology (GO) enrichment analysis is a widely used bioinformatics tool for interpreting high-throughput data. The three main aspects of the GO classification system are molecular function (MF), cellular composition (CC), and biological processes (BP). The Kyoto Encyclopedia of Genes and Genomes (KEGG) is an information repository that promotes a comprehensive examination of gene function by linking genomic data with signaling pathways, contributing to explorations of the mechanisms of diseases. The “clusterProfiler” R package was employed for the GO and KEGG pathway analyses performed in this study. Significantly enriched pathways were selected based on a *P*-value < 0.05.

### Assessment of immune cell proportions

The CIBERSORT analytical tool was utilized to determine the composition of 22 types of immune cells in each sample. Subsequently, a comparison was made between the HCC and LCC groups to examine the relative proportions of these immune cells. The “corrplot” R package was used to perform the correlation analyses.

### Construction of a ceRNA network

The mRNA–miRNA interactions for PRKAR2B were predicted using the starBase database. The miRNA–lncRNA interactions were then screened to obtain the ceRNA network.

### Statistical analysis

The data analysis was conducted using R software (Version 4.2.2). Student's *t*-test was used for comparisons between two groups. Visualization of the ceRNA network was achieved using Cytoscape (Version 3.10.0).

## Results

### Identification of three hub features in patients with capsular contracture

**Figure 1:**
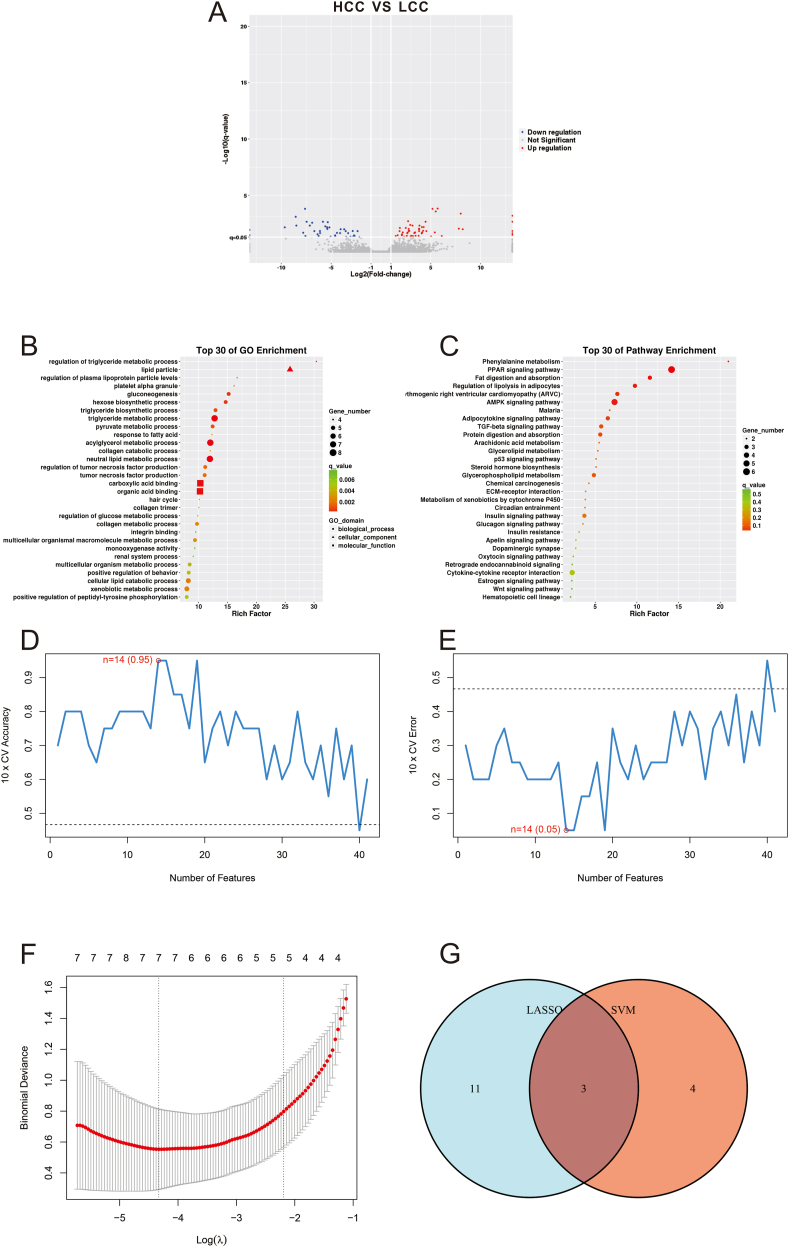
Identification of hub features in patients with capsular contracture. **(A)** A volcano plot of the differential gene expression. **(B)** The top 30 enriched GO terms. **(C)** The top 30 KEGG pathway enrichment terms. **(D, E)** The true and error value was calculated by the SVM-RFE algorithm; 14 diagnostic genes were identified. **(F)** Seven diagnostic genes were predicted by the LASSO logistic regression algorithm. **(G)** A Venn diagram of the diagnostic genes identified by the two algorithms; three diagnostic markers were screened from the two algorithms. Blue represents the SVM-RFE results; orange represents the LASSO results. CC, capsular contracture; SVM, support vector machine; RFE, recursive feature elimination; LASSO, least absolute shrinkage and selection operator.

A total of 93 genes exhibited differential expression between the HCC and LCC groups in the cellular composition, meeting the screening criteria. A volcano plot (Fig. 1A) demonstrating the top 30 enriched GO terms (*P*_*adjust*_ < 0.05) is presented in Figure 1B. These GO terms included regulation of the triglyceride metabolic process, lipid particles, regulation of plasma lipoprotein particle levels, platelet alpha granule, gluconeogenesis, and the hexose biosynthetic process, among others. Figure 1C shows the top 30 enriched KEGG pathways (*P*_*adjust*_ < 0.05). These pathways encompassed phenylalanine metabolism, the PPAR signaling pathway, fat digestion and absorption, regulation of lipolysis in adipocytes, and adipocytokine signaling pathway, among others. The DEGs exhibited significant enrichment in functions and pathways related to lipid metabolism. These pathways are thus considered to be associated with the pathogenic development and/or progression of breast capsular contracture. Consequently, 41 genes related to lipid metabolism were screened in further investigations (Table S1). Two different machine learning algorithms (SVM-RFE and LASSO) were utilized to assess the diagnostic value of the DEGs. Fourteen features related to lipid metabolism were screened using the SVM-RFE algorithm [maximal accuracy = 0.95 (Fig. 1D), minimal RMSE = 0.05 (Fig. 1E)]. Then, the LASSO algorithm was applied separately and identified seven characteristic genes (Fig. 1F). The intersecting genes (FGF7, PRKAR2B, and FAM135B) were classed as candidate diagnostic value features (Fig. 1G).

### Two types of immune cells are differentially present between the HCC and LCC groups

**Figure 2:**
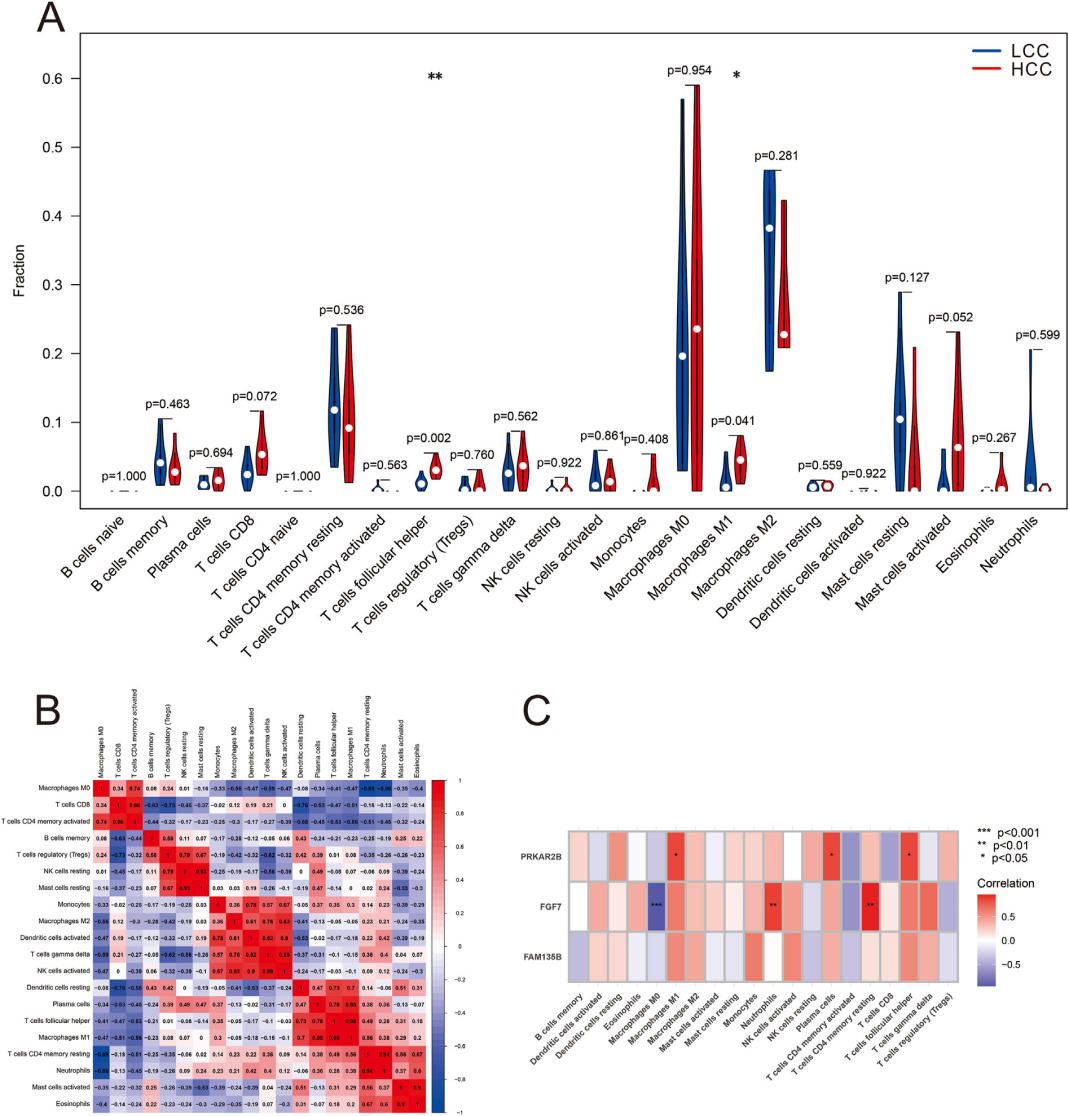
Immune cell infiltration analysis. **(A)** Visualization of the expression level of 22 types of immune cells present in HCC and LCC samples. The proportions of follicular helper T cells and M1 macrophages were different between the two groups. **(B)** A correlation heatmap of the 22 immune cell subtypes. **(C)** A correlation analysis between the diagnostic genes and 22 immune cell subtypes. The correlations of PRKAR2B with follicular helper T cells and M1 macrophages. CC, capsular contracture. ^∗^*P* < 0.05, ^∗∗^*P* < 0.01, ^∗∗∗^*P* < 0.001.

We employed the CIBERSORT method to investigate differences in the immune microenvironment between the LCC and HCC groups. Our analysis revealed significant differences in two types of immune cells, M1 macrophages (*P* < 0.05) and follicular helper T cells (*P* < 0.01) (Fig. 2A). These two cell types exhibited higher proportions in the HCC group than in the LCC group. Furthermore, we examined the correlations among the 22 immune cell types (Fig. 2B). Notably, we observed several datasets with highly correlated functions. Specifically, we found a strong positive correlation between M1 macrophages and follicular helper T cells (*r* = 0.98). Additionally, M1 macrophages showed a highly positive correlation with plasma cells (*r* = 0.85) and resting dendritic cells (*r* = 0.70). Follicular helper T cells also exhibited a highly positive correlation with plasma cells (*r* = 0.78) and resting dendritic cells (*r* = 0.73). We also observed a negative correlation between M1 macrophages and activated CD4 memory T cells (*r* = −0.56), as well as between follicular helper T cells and activated CD4 memory T cells (*r* = −0.53). Thus, our analysis revealed significant differences in the immune microenvironment between the LCC and HCC groups. Moreover, we identified strong correlations among several immune cell types, both positive and negative, highlighting their potential interactions in the context of the study.

### Positive correlation between PRKAR2B and the differential immune cells

**Figure 3:**
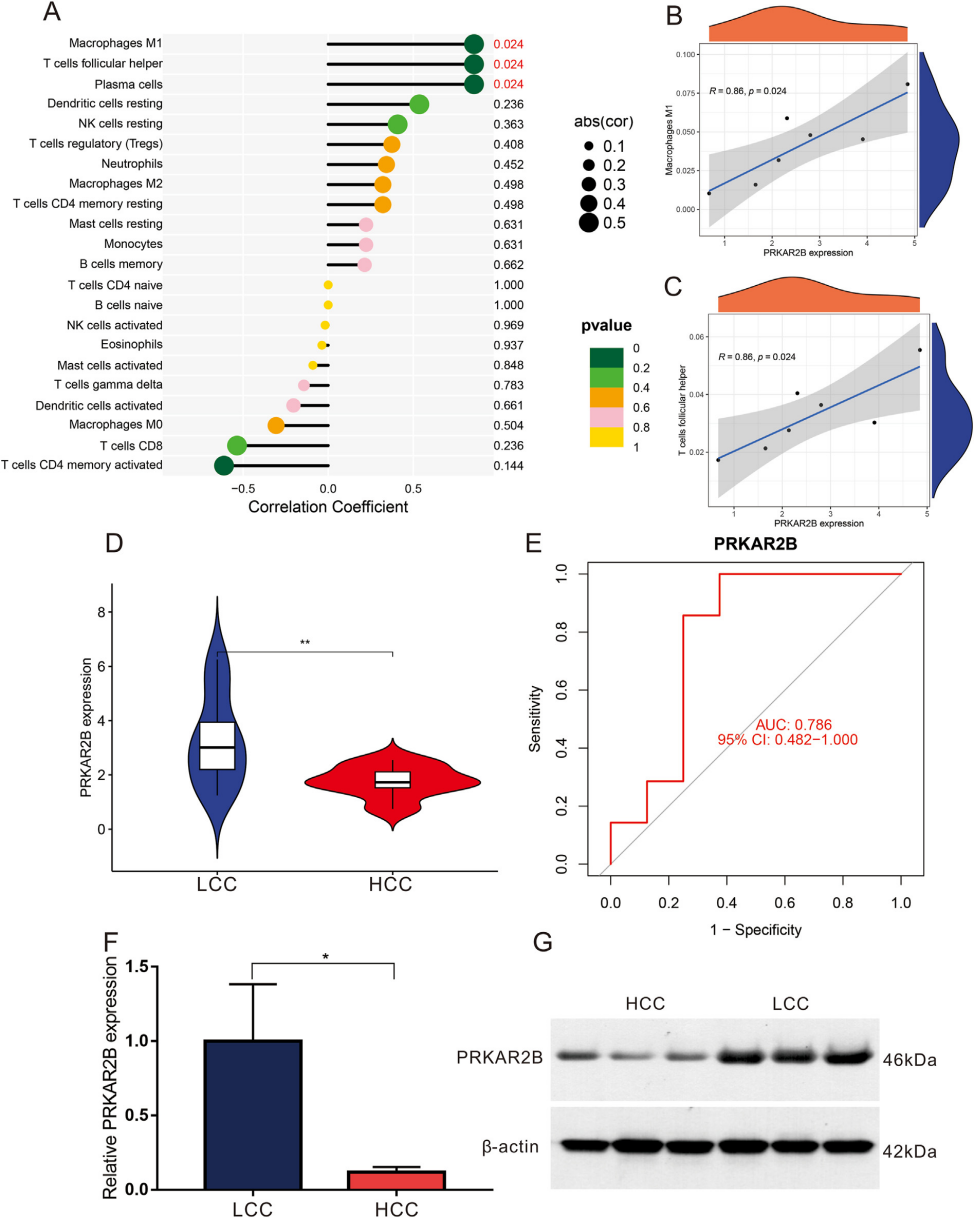
Correlation analysis between PRKAR2B and infiltrating immune cells, and the diagnostic value of PRKAR2B. **(A)** Visualization of the correlation analysis between PRKAR2B and 22 types of immune cells. **(B)** A scatter diagram showing the correlation between PRKAR2B expression and follicular helper T cells. **(C)** A scatter diagram showing the correlation between PRKAR2B expression and M1 macrophages. The performance of PRKAR2B to diagnose CC. **(D)** The differences in PRKAR2B expression between the two groups determined by RNA-seq. **(E)** The receiver operating characteristic curve of PRKAR2B in the two groups. **(F)** The differences in PRKAR2B expression between the two groups determined by qRT-PCR. **(G)** The differences in PRKAR2B protein expression between the two groups determined by Western blot (WB). CC, capsular contracture; qRT-PCR, real-time quantitative polymerase chain reaction. ^∗^*P* < 0.05, ^∗∗^*P* < 0.01.

To confirm the value of candidate diagnostic genes, we further analyzed the relationship between candidate feature genes and the immune microenvironment by a Pearson analysis. The results revealed that PRKAR2B was positively correlated with M1 macrophages (*P* < 0.05), plasma cells (*P* < 0.05), and follicular helper T cells (*p* < 0.05). FGF7 had strong positive and negative correlations with M0 macrophages (*P* < 0.001), neutrophils (*P* < 0.01), and resting CD4 memory T cells (*P* < 0.01) (Fig. 2C). We next explored the correlation between PRKAR2B and each type of immune cell (Fig. 3A). The visualization indicated that PRKAR2B was positively correlated with M1 macrophages (*r* = 0.86, *P* < 0.05; Fig. 3B) and follicular helper T cells (*r* = 0.86, *P* < 0.05; Fig. 3C).

### Prognostic value of PRKAR2B

As expected, the expression of PRKAR2B was found to be lower in the HCC group (*P* < 0.01; Fig. 3D). The result was validated in subsequent experiments (Fig. 3F, G). The diagnostic effectiveness of PRKAR2B for capsular contracture was examined by a ROC curve. The AUC was 0.786 (95% CI: 0.482–1.000; Fig. 3E), suggesting that it has good effectiveness for the diagnosis.

**Figure 4:**
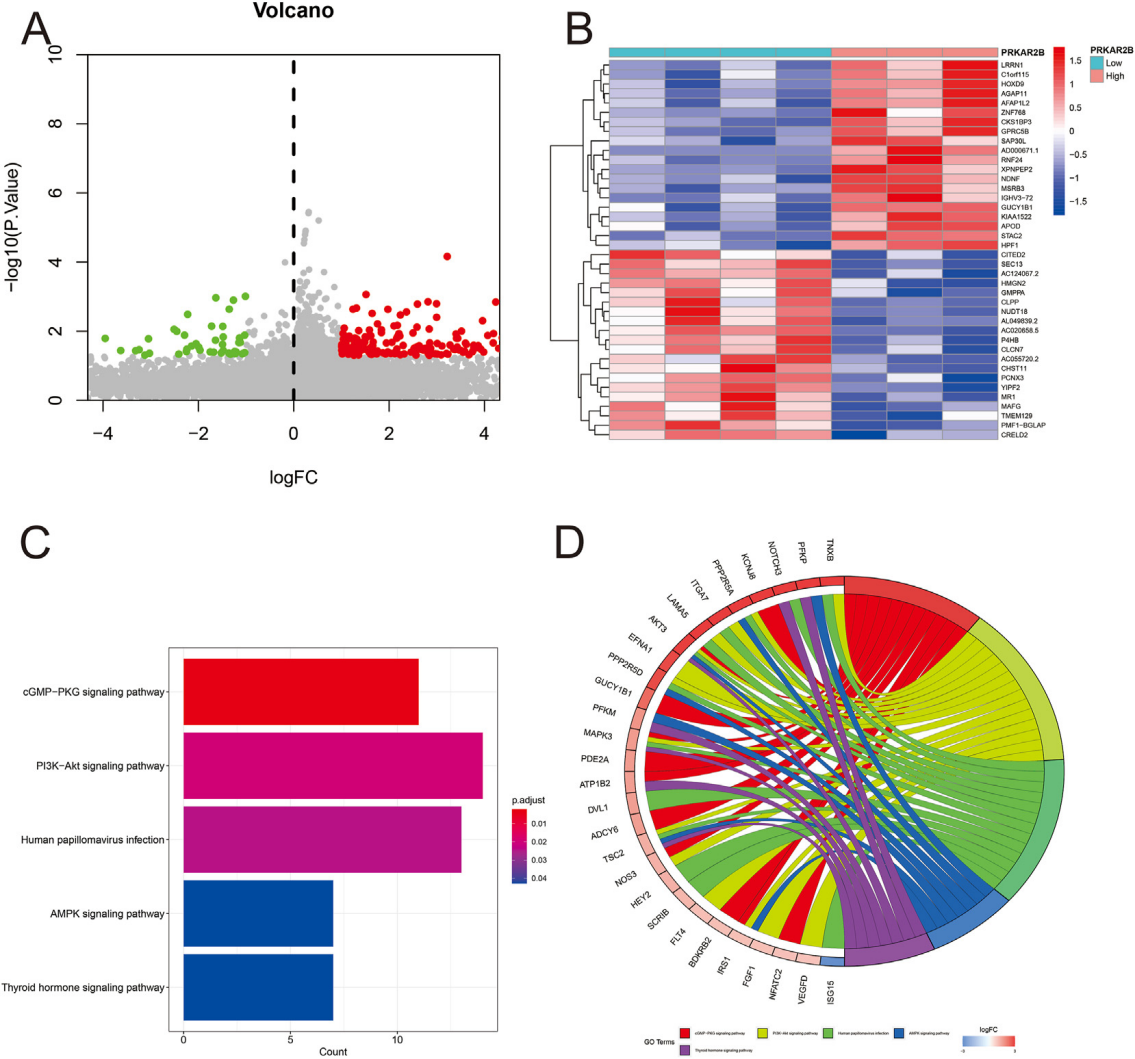
Functional enrichment analysis of differential gene expression between the high and low PRKAR2B expression groups. **(A)** A volcano plot showing the differential gene expression. **(B)** A heatmap of the top 20 differentially (high–low) expressed genes. **(C)** A bar plot of the enriched KEGG terms. **(D)** A chord diagram of the enriched KEGG terms. High, PRKAR2B high expression group; Low, PRKAR2B low expression group.

Finally, we used PRKAR2B as a biomarker gene. We categorized the capsular contracture samples into two groups, high PRKAR2B (above median expression level) and low PRKAR2B (below median expression level) expression groups. There were 271 DEGs between the two groups (Fig. 4A). Figure 4B illustrates the top 20 (high–low) DEGs. A KEGG pathway enrichment of the DEGs was conducted and the results are depicted as a bar plot (Fig. 4C). The enriched genes were main in the cGMP−PKG signaling pathway, PI3K−Akt signaling pathway, human papillomavirus infection, AMPK signaling pathway, and thyroid hormone signaling pathway. A total of 28 DEGs were noted in the KEGG pathway enrichment analysis (Fig. 4D).

### ceRNA networks based on PRKAR2B

**Figure 5:**
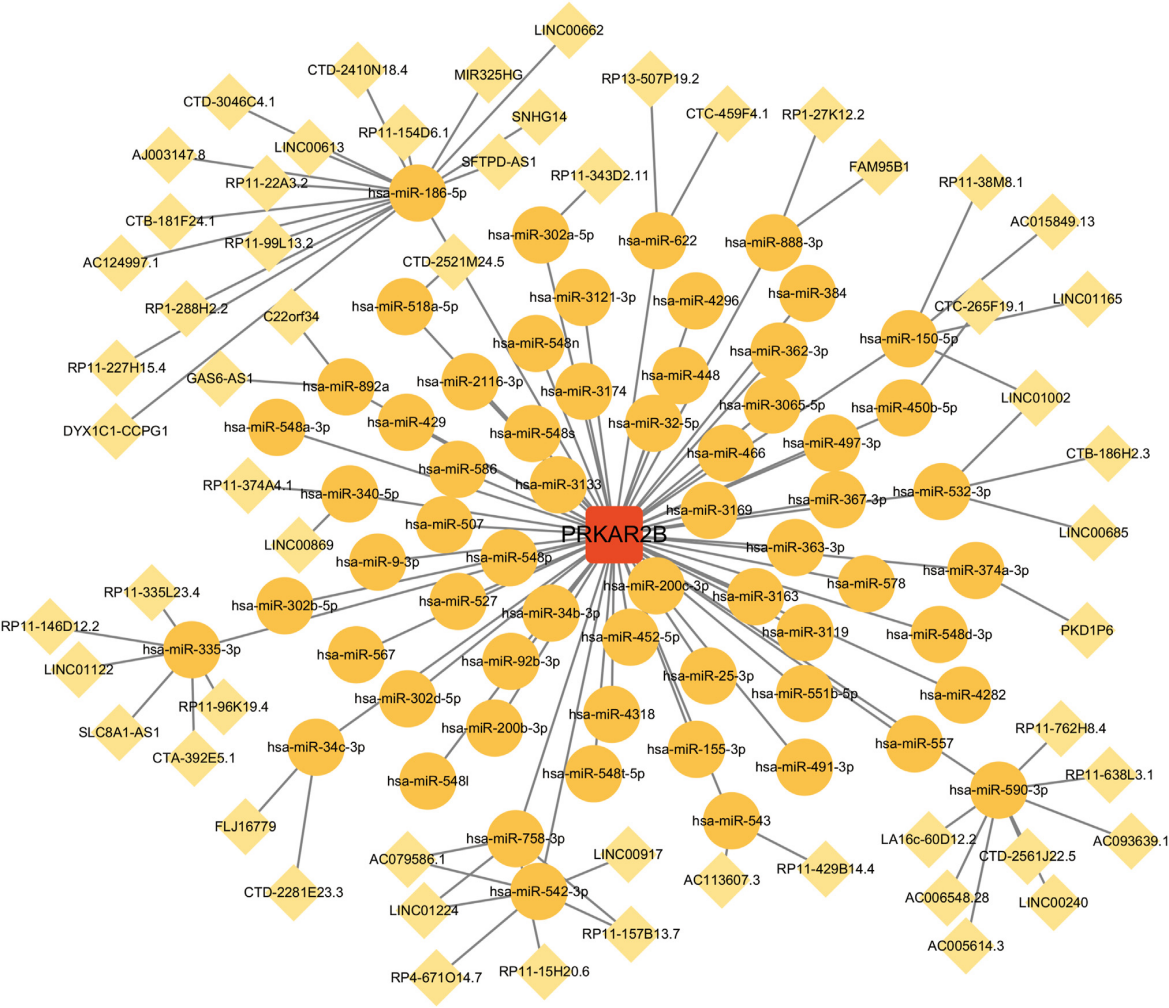
A ceRNA network based on PRKAR2B. Red represents the diagnostic genes, orange represents miRNAs, and yellow represents lncRNAs. miRNAs, microRNAs; lncRNAs, long non-coding RNAs.

A ceRNA network was constructed based on PRKAR2B, which included 121 nodes (1 diagnostic gene, 62 miRNAs, and 58 lncRNAs) and 124 edges (Fig. 5). The findings support that PRKAR2B may be useful as a diagnostic marker for PRKAR2B and suggest possible mechanistic pathways involved in the development of the condition.

## Discussion

Capsular contracture is a common and severe complication after breast prosthesis implantation,^19^ and its diagnosis and grading are predominantly based on the Baker grading system, which is a subjective system. The identification of biological markers of diseases using machine learning algorithms has been performed for many diseases, such as coronary artery disease,^20^ diabetic nephropathy,^21^ and essential thrombocythemia.^22^ It is reasonable to consider DEGs as possible prognostic markers for the clinical classification of breast capsular contracture. Here, we identified PRKAR2B as a new biomarker for breast capsular contracture.

To establish biological diagnostic features for breast capsular contracture, we initially identified 41 lipid metabolism-related genes through a biological function enrichment analysis. Subsequently, we employed two distinct algorithms, SVM-RFE and LASSO, on the 41 DEGs. This analysis highlighted three intersecting genes — FGF7, FAM135B, and PRKAR2B — as potential candidate genes for further investigation.

Considering the correlation between capsular contracture and the chronic inflammatory response mediated by macrophages, it is not surprising that our analysis of immune infiltration revealed higher infiltration of two types of immune cells in the HCC group. This observation suggests that capsular contracture increases with an enhanced inflammatory response (M1 macrophages), aligning with previous research findings.^23^ In addition, we found a positive correlation between PRKAR2B and M1 macrophages, and between PRKAR2B expression and follicular helper T cells. Dong et al^27^ also found that M1 macrophages were positively correlated with PRKAR2B. However, to our knowledge, the present study is the first to show a correlation between PRKAR2B expression and follicular helper T cells. Therefore, PRKAR2B was regarded as a potential biological marker for breast capsular contracture, supported by its diagnostic value indicated by the Area Under Curve (AUC) value (0.786).

According to recent reports,^24^ PRKAR2B is involved in the process of bioenergy metabolism. Kerr et al^25^ evaluated the impact of specific genes on lipid storage based on siRNA-mediated silencing. They found that PRKAR2B knockdown affected lipid metabolism and storage. PRKAR2B was also discovered to enhance prostate cancer metastasis by activating the Wnt/β-catenin signaling pathway.^26^ Some studies suggested that PRKAR2B can serve as a potential biological marker for diabetic kidney disease^27^, ^28^, ^29^, ^30^ and squamous cell carcinoma.^31^ PRKAR2B has been found to be related to various other diseases. For example, a higher level of PRKAR2B was observed in the cartilage of patients with osteoarthritis compared with those with acute or chronic instability.^32^ PRKAR2B has also been linked to the diagnosis/prognosis of type 2 diabetes,^33^ chronic obstructive pulmonary disease,^34^ and hypertension.^35^

To investigate the mechanism(s) underlying breast capsular contracture, we categorized the samples of capsular contracture into two groups. Subsequently, a KEGG enrichment analysis identified five signaling pathways that exhibited significant enrichment among the DEGs between the two groups. Some scholars have reported that there is an association of the cGMP-PKG signaling pathway with lung fibrosis,^36^ and the PI3K-Akt signaling pathway with pulmonary fibrosis,^37^^,^^38^ cardiac fibrosis,^39^ fibrosis of chronic kidney disease,^40^ and liver fibrosis.^41^ The thyroid hormone signaling pathway has also been implicated in fibrotic diseases.^42^, ^43^, ^44^ We found that these molecular pathways may also be associated with fibrosis related to capsular contracture. Finally, we constructed a ceRNA network based on PRKAR2B, which might provide novel biomarkers for breast capsular contracture.

In summary, our study demonstrated that PRKAR2B is a novel diagnostic biomarker for breast capsular contracture and might have applications in a new grading system for this complication of breast augmentation surgery. The cGMP-PKG, PI3K-Akt, and thyroid hormone signaling pathways are considered to be involved in the development of breast capsular contracture.

## Ethics declaration

Ethical approval was obtained for this study from the Plastic Surgery Hospital Ethics Committee. All patients signed informed consent before participating.

## Author contributions

JL and SF conceived and designed the study strategy. YM and XH performed the literature search and collected study data and references. YM prepared figures and tables. YM and XH drafted the manuscript. JL and SF revised the manuscript. All the authors read and approved the final version of the manuscript.

## Conflict of interests

The authors declare that there is no conflict of interests.

## Funding

This work was supported by the key projects of medical school development of Shijingshan District, Beijing, China (No. 20078) and the National Clinical Key Specialty Construction Project (China) (1112621048).
