# Supplementary material for: Transcriptomic and machine learning analyses identify hub genes of metabolism and host immune response that are associated with the progression of breast capsular contracture
Source: Genes Dis. 2023 Sep 9;11(3):101087. doi: 10.1016/j.gendis.2023.101087 (PMC10825289; doi:10.1016/j.gendis.2023.101087)
Supplement: Multimedia component 1 [file mmc1.docx]

Table S1. Clinical characteristics of patients.

|  | LCC group | HCC group | All |
| --- | --- | --- | --- |
| Sample size | N=8(6 patients) | N=7(6 patients) | N=15(12patients) |
| Gender(Male/Female) | 0/6 | 0/6 | 0/12 |
| Implant layer(pre/subpectoral) | Subpectoral | Subpectoral | Subpectoral |
| Capsular contracture(Ⅰ-Ⅳ) | ⅠandⅡ | Ⅲ and Ⅳ | Ⅰ-Ⅳ |
