# Supplementary material for: Transcriptomic and machine learning analyses identify hub genes of metabolism and host immune response that are associated with the progression of breast capsular contracture
Source: Genes Dis. 2023 Sep 9;11(3):101087. doi: 10.1016/j.gendis.2023.101087 (PMC10825289; doi:10.1016/j.gendis.2023.101087)
Supplement: Multimedia component 2 [file mmc2.docx]

Table S2. Genes related to lipid metabolism.

| Gene |
| --- |
| THRSP  AADAC  GPAM  DGAT2  PLIN1  CIDEA  PLIN4  CIDEC  LIPE  PCK1  GPD1  CYP26A1  CYP4F3  ADIPOQ  PLA2G2A  LGALS12  NR4A3  FGF7  THBS1  INHBA  CD36  AKR1C1  PRKAR2B  AKR1C2  FAM135B  STC2  CX3CR1  TAT  TNFRSF11B  PFKFB1  MGST1  TIMP4  GAS6  MRAP  GLYAT  AQP7  FST  DKK2  RSPO2  NAT8L  MMP3 |
