# Supplementary material for: Transcriptomic and machine learning analyses identify hub genes of metabolism and host immune response that are associated with the progression of breast capsular contracture
Source: Genes Dis. 2023 Sep 9;11(3):101087. doi: 10.1016/j.gendis.2023.101087 (PMC10825289; doi:10.1016/j.gendis.2023.101087)
Supplement: Multimedia component 3 [file mmc3.docx]

Table S3. List of primers used in the study.

| Gene | Forward primer | Reverse primer |
| --- | --- | --- |
| GAPDH | ACAGCCTCAAGATCATCAGCAAT | GATGGCATGGACTGTGGTCAT |
| PRKAR2B | AGTATGGTTCTGTATGGCAATAGGT | CCTAGTTAGCCAGATTGATTTGGT |
